# Increased CD8+CD28+ T cells independently predict better early response to stereotactic ablative radiotherapy in patients with lung metastases from non-small cell lung cancer

**DOI:** 10.1186/s12967-019-1872-9

**Published:** 2019-04-11

**Authors:** Chao Liu, Qinyong Hu, Kai Hu, Huichao Su, Fang Shi, Li Kong, Hui Zhu, Jinming Yu

**Affiliations:** 1 Department of Oncology, Renmin Hospital of Wuhan University, Wuhan, 430060 China; 2 Department of Radiation Oncology, Shandong Cancer Hospital and Institute, Shandong Cancer Hospital Affiliated to Shandong University, Shandong Academy of Medical Sciences, Jinan, 250117 Shandong China; 3 Department of Radiation Oncology, Affiliated Hospital of Academy of Military Medical Sciences, Beijing, 100071 China; 4 Department of Radiation Oncology, The First Affiliated Hospital of Guangxi Medical University, Nanning, 530021 Guangxi China

**Keywords:** Lung metastases, Stereotactic ablative radiotherapy, Tumor response, Biomarker, Immunology

## Abstract

**Background:**

Stereotactic ablative radiotherapy (SABR) shows a remarkable local control of non-small cell lung cancer (NSCLC) metastases, partially as a result of host immune status. However, the predictors of immune cells for tumor response after SABR are unknown. To that effect, we investigated the ability of pre-SABR immune cells in peripheral blood to predict early tumor response to SABR in patients with lung metastases from NSCLC.

**Methods:**

This study included 70 patients with lung metastases from NSCLC who were undergoing SABR. We evaluated the early tumor response 1 month and 6 months after SABR in these patients following RECIST 1.1 guidelines. Pre-SABR peripheral CD8+ T cell count, CD8+CD28+ T-cell count, CD8+CD28− T-cell count, CD4+ T-cell count, and Treg-cell count were measured using flow cytometry.

**Results:**

Increased CD8+CD28+ T-cell counts (14.43 ± 0.65 vs. 10.21 ± 0.66; P = 0.001) and CD4/Treg ratio (16.96 ± 1.76 vs. 11.91 ± 0.74; P = 0.011) were noted in 1-month responsive patients, compared with non-responsive patients. In univariate logistic analyses, high CD8+CD28+ T-cell counts (OR 0.12, 95% CI 0.03–0.48; P = 0.003), CD4/Treg ratio (OR 0.24, 95% CI 0.06–0.90; P = 0.035), and BED_10_ (OR 0.91, 95% CI 0.84–0.99; P = 0.032) predicted a 1-month tumor response to SABR. According to multivariate logistic analyses, the CD8+CD28+ T-cell count predicted a 1-month tumor response to SABR (OR 0.19, 95% CI 0.04–0.90; P = 0.037) independently. Furthermore, we confirmed the independent predictive value of the CD8+CD28+ T-cell count in predicting tumor response to SABR in 41 patients 6 months after treatment (OR 0.08, 95% CI 0.01–0.85; P = 0.039).

**Conclusions:**

A pre-SABR CD8+CD28+ T-cell count could predict early tumor response to SABR in patients with lung metastases from NSCLC. Larger, independently prospective analyses are warranted to verify our findings.

**Electronic supplementary material:**

The online version of this article (10.1186/s12967-019-1872-9) contains supplementary material, which is available to authorized users.

## Background

Among malignant tumors, lung cancer is a leading global cause of death due to its aggressive tumor evasion and metastasis characteristics [1, 2]. Surgery is generally regarded as the standard of care for patients with early-stage and oligometastatic non-small cell lung cancer (NSCLC) [3–6]. Recently, stereotactic ablative radiotherapy (SABR), a high-precision treatment approach that combines multiple technological advancements for the delivery of radiation, has become increasingly useful as a significant alternative therapy for patients with early-stage and oligometastatic NSCLC who are at high risk of various surgical complications [7–13]. Notably, the effectiveness of SABR for early-stage NSCLC is comparable to that of surgery. A pooled analysis of two independent, randomized, phase III trials showed that overall rates of 3 years survival were 95% in the SABR group and 79% in the surgery group for operable stage I NSCLC [14]. Additionally, for oligometastatic NSCLC and lung metastases, SABR showed remarkable efficiency with regard to local control and survival [11–13, 15]. Lodeweges et al. [15], meanwhile, reported 5-year overall survival rates of 41% for surgery and 45% for SABR in patients with pulmonary oligometastases.

Despite SABR’s remarkable control of local NSCLC lesions, patients have shown mixed early tumor responses. However, markers to predict early tumor response to SABR have not been investigated thoroughly. A previous study revealed that at least a 20% lung lesion shrinkage by the final session of SABR could be predictive of a complete response within 6 months [16]. Also, Mazzola et al. [17] reported the mean and maximum values of pre-SABR standard uptake value to be both significantly correlated with a complete response within 6 months after SABR treatment of lung metastases from various primary tumors.

Multiple parameters, such as occurrence, development, recurrence, and metastasis of tumors, comprise processes by which tumors evade immune surveillance; this evasion is closely related to host immune function. Many studies have investigated the predictive values of peripheral and tumor-infiltrating lymphocyte (TIL) subsets to assess tumor response to chemotherapy, radiotherapy, and chemo-radiotherapy in various tumors [18–23]. For example, several previous studies have revealed significant correlations between a variety of parameters (e.g., CD4+ TILs, CD8+ TILs, tumor-infiltrating myeloid-derived suppressor cells, and peripheral lymphocyte number) and the tumor response to neoadjuvant chemo-radiotherapy for advanced rectal cancer [19, 24]. In a study of breast cancer patients, the TILs and PD-L1 assessed in the epithelium or stroma were predictive of a complete pathological response to neoadjuvant chemotherapy [21]. Additionally, peripheral CD8+ T-cell counts, CD3+ T-cell counts, CD19+ B-cell counts, and CD4/CD8 ratio all showed relationships with tumor response to carbon ion radiotherapy in patients with prostate cancer [25].

The activation of CD8+ T cells involves both the T cell receptor (TCR) and CD28 signals [26, 27]. As an essential co-stimulatory molecule, CD28 on CD8+ T cells interacts with B7 molecules on antigen-presenting cells to activate the anti-tumor immune response of CD8+ T cells to tumor antigens. However, CD8+ T cells in cancer patients can lose the expression of CD28 due to the chronic stimulation of tumor antigens and consequently present with a non-responsive status to tumor antigens [28, 29]. We also reported this phenomenon in our previous study, as did other studies, that decreased CD8+CD28+ T cells and increased CD8+CD28− T cells were observable in NSCLC patients when compared with healthy volunteers [30–32]. In two recent studies, PD-1 inhibited the function of T cells by inactivating CD28 signaling, and PD-1-targeted therapies rescued CD28+ cells but not CD28− cells among CD8+ T cells, suggesting that CD28 signal plays vital roles in regulating the function of effector T cells [33, 34]. Thus, CD8+CD28+ T cells may exert anti-tumor efficiency among CD8+ T cells.

More importantly for SABR, a growing number of studies have shown that its remarkable efficiency is partially a result of host immune status and the interaction between SABR and the immune response [35–37]. Specifically, SABR could facilitate the immunogenic cell death of cancer cells, release tumor antigens, recruit antigen-presenting cells to present antigens to T cells, and activate the antitumor effect of CD8+ T cells through TCR and CD28 signals [38]. Thus, we speculate that the CD8+CD28+ T-cell count is associated with the response to SABR in patients. However, thus far, no previous study has examined the predictive value of immune factors for early tumor response to SABR in patients with lung metastases. Therefore, our aim consisted of evaluating the predictive roles of pre-SABR CD8+ T-cell counts, CD8+CD28+ T-cell counts, CD8+CD28− T-cell counts, CD4+ T-cell counts, and Treg-cell counts in peripheral blood for early tumor response to SABR in patients with lung metastases from NSCLC.

## Methods

### Patient selection

A total of 70 patients with histologically confirmed lung metastases from NSCLC, who were treated with SABR between January 2015 and September 2018, were included in the present study. All patients received definitive treatment for primary tumors. We excluded patients (1) aged < 18 years; (2) with performance status > 2; (3) who received an anti-tumor treatment or steroids during the 3 months leading to enrollment; (4) who received concurrent chemotherapy, targeted therapy, or other anti-tumor treatment within 1 month leading to SABR; (5) with other malignant tumors; (6) with hematonosis; (7) with immune diseases, including rheumatoid arthritis, systemic lupus erythematosus, chronic liver disease, ulcerative colitis, hyperthyroidism, and scleroderma; and/or (8) with renal diseases. The Ethics Committee of the Affiliated Hospital of the Academy of Military Medical Sciences approved this study. All patients and volunteers provided written informed consents.

Patient characteristics, including sex, age, primary T stage, primary N stage, primary AJCC stage (based on AJCC-7 criteria [39]), histology, performance status, and smoking history, were collected from electronic medical records.

### Detection of lung metastases and SABR

Lung metastases were identified by tumor biopsies, as well as by computed tomography (CT) and positron emission tomography/computed tomography (PET/CT). All lung metastases were treated with SABR using a CyberKnife. Respiratory-induced tumor motion was tracked using a real-time tumor tracking system. The prescribed radiation therapy dose was administered at the discretion of the treating radiation oncologist, in order to respect normal tissue tolerances. Fifty gray in 5 fractions, or 70 gray in 10 fractions, were used. BED_10_ was assessed using the formula D × [1 + d/(α/β)], where D is the total dose, d is the dose per fraction, and α/β is 10 [40].

### Early tumor response to SABR

We evaluated the early tumor response 1 month and 6 months after SABR treatment per RECIST guidelines (version 1.1) [41] by using CT or PET-CT. The disappearance of targeted lung metastases characterized a complete response (CR). A partial response (PR) was characterized by a reduction of at least 30% of the diameter of targeted lung metastases. Progressive disease was characterized by an increase of at least 20% of the diameter of targeted lung metastases. Stable disease (SD) was characterized by the absence of sufficient shrinkage to meet the definition for PR or the lack of sufficient increase to meet the definition for PD. One month after SABR, patients who exhibited CR and PR were labelled responsive patients, while those who exhibited SD and PD were referred to as non-responsive patients. Furthermore, patients who exhibited CR were denoted responsive patients, and those who exhibited PR and SD were referred to as non-responsive patients 6 months after SABR; since CR was observed in almost half of the patients at this time.

### Flow cytometry

**Fig. 1:**
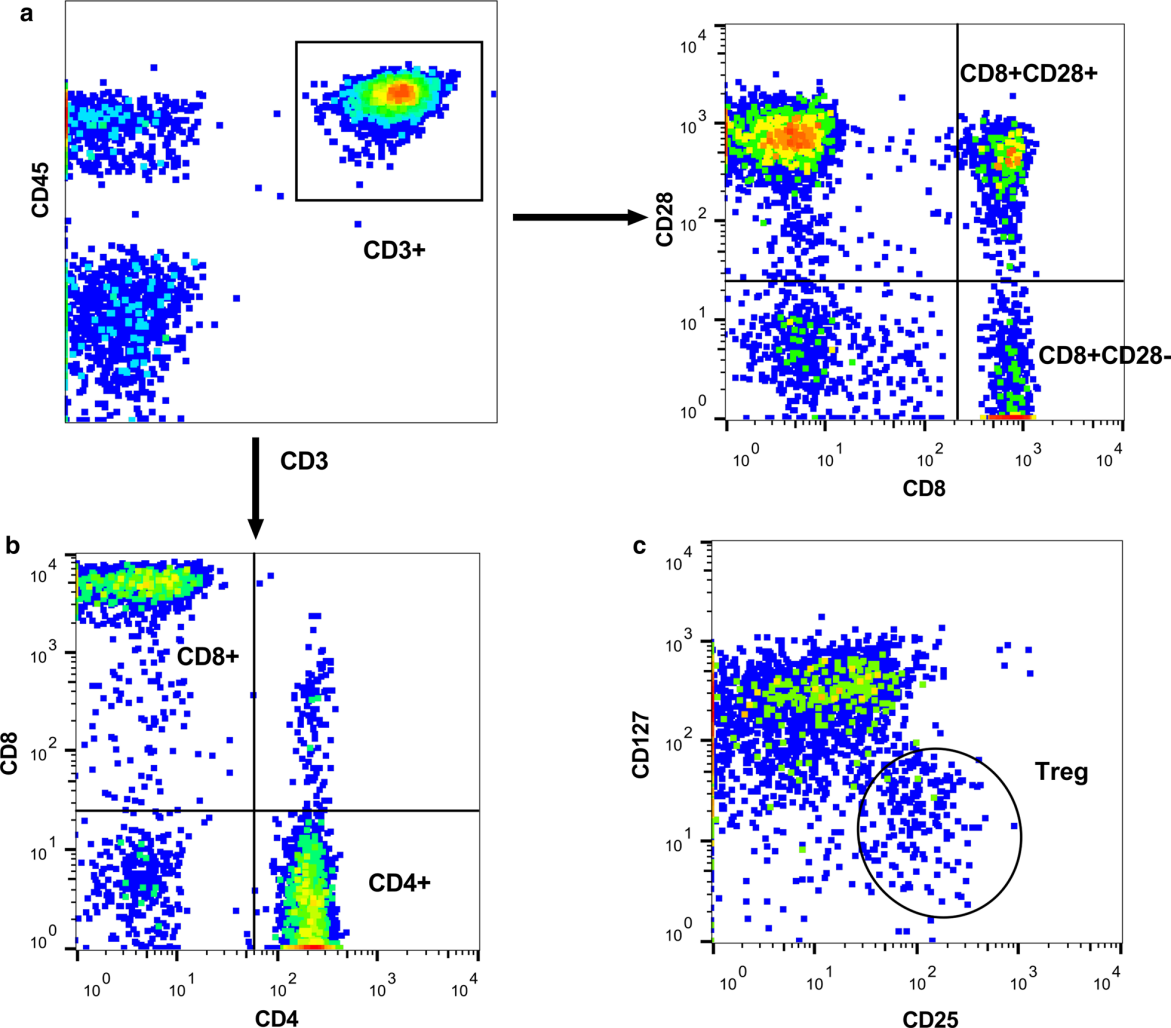
Typical flow cytometry plots and gating for **a** CD8+CD28+ and CD8+CD28− T cells; **b** CD8+ and CD4+ T cells; and **c** Treg cells

The protocol for flow cytometry has been described in our previous study [30]. Four milliliters of fresh blood were collected and stored in EDTA anti-coagulate tubes within 7 days before SABR. CD8+ T-cell count (CD3+CD8+CD4−), CD8+CD28+ T-cell count (CD3+CD8+CD28+), CD8+CD28− T-cell count (CD3+CD8+CD28−), CD4+ T-cell count (CD3+CD4+CD8−), and Treg-cell count (CD4+CD25+CD127^low^) were assessed. Figure 1 shows representative flow cytometry plots and gating.

### Statistical analysis

Data were evaluated using the SPSS 23.0 software (SPSS Inc., Chicago, IL, USA). A receiver operating characteristic (ROC) curve was used to determine high and low immune cells to distinguish between responsive and non-responsive patients. The independent Student’s t-test was used for comparison of differences involving immune cells between responsive and non-responsive patients. Logistic regression was used to assess the relationships between factors and early tumor response after SABR. Variables with *P *< 0.05 in univariate analyses were used in multivariate analyses. A *P* value < 0.05 was considered to be statistically significant.

## Results

### Patient characteristics

**Fig. 2:**
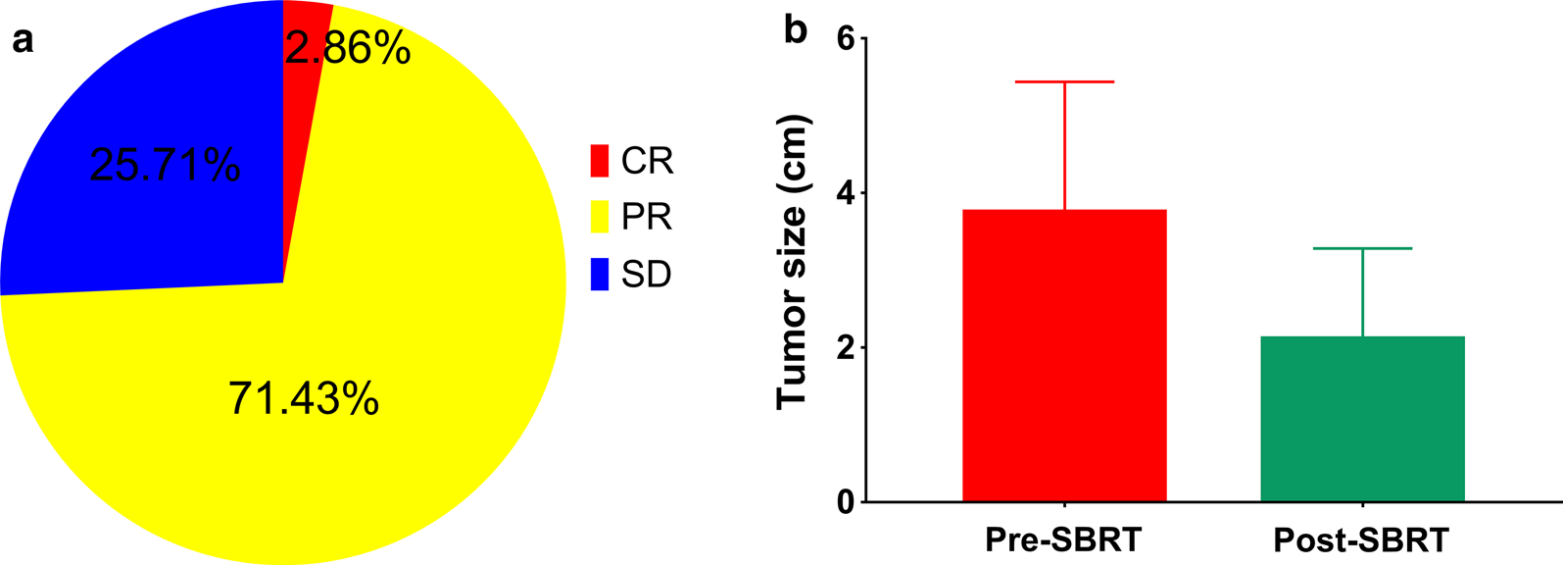
Tumor response 1 month after SABR. **a** Pie chart of tumor response (CR, PR, and SD); **b** Changes in tumor size after SABR

Table 1 presents the clinicopathological characteristics of 70 enrolled patients. The median age was 64 (44–90) years. Fifty-two (74.3%) patients had isolated lung metastases, while 18 (25.7%) patients had multiple metastases. The median diameter of targeted lung metastases was 3.5 (1.3–7.9) cm. Based on the RECIST 1.1 guidelines, 2 (2.86%) patients experienced CR, 50 (71.43%) experienced PR, and 18 (25.71%) experienced SD, 1 month after SABR (Fig. 2a); the mean tumor size of lung metastases decreased from 3.75 ± 0.24 to 2.11 ± 0.17 cm (Fig. 2b). Fourty-one patients were evaluated for tumor response 6 months after SABR; 18 (43.90%) patients experienced CR, 19 (46.34%) experienced PR, and 4 (9.75%) experienced SD.

**Table 1:** Clinicopathological characteristics of 70 patients with lung metastases from NSCLC

Factors	N	%
Sex		
Male	47	67.1
Female	23	32.9
Median age	64 (44–90)	
Primary T stage		
T1	20	28.6
T2	30	42.9
T3	10	14.3
T4	10	14.3
Primary N stage		
N0	22	31.4
N1	20	28.6
N2	19	27.1
N3	9	12.9
Primary stage		
I	15	21.4
II	19	27.1
III	36	51.4
Histology		
SCC	38	54.3
AD	32	45.7
Performance status		
0	33	47.1
1	36	51.4
2	1	1.4
Smoking history		
Smoker	42	60.0
Non-smoker	28	40.0
Metastatic status		
Isolated lung metastasis	52	74.3
Multiple metastases	18	25.7
The diameter of targeted lung metastases	3.5 (1.3–7.9) cm

### Increased CD8+CD28+ T-cell count and CD4/Treg ratio in responsive patients

**Fig. 3:**
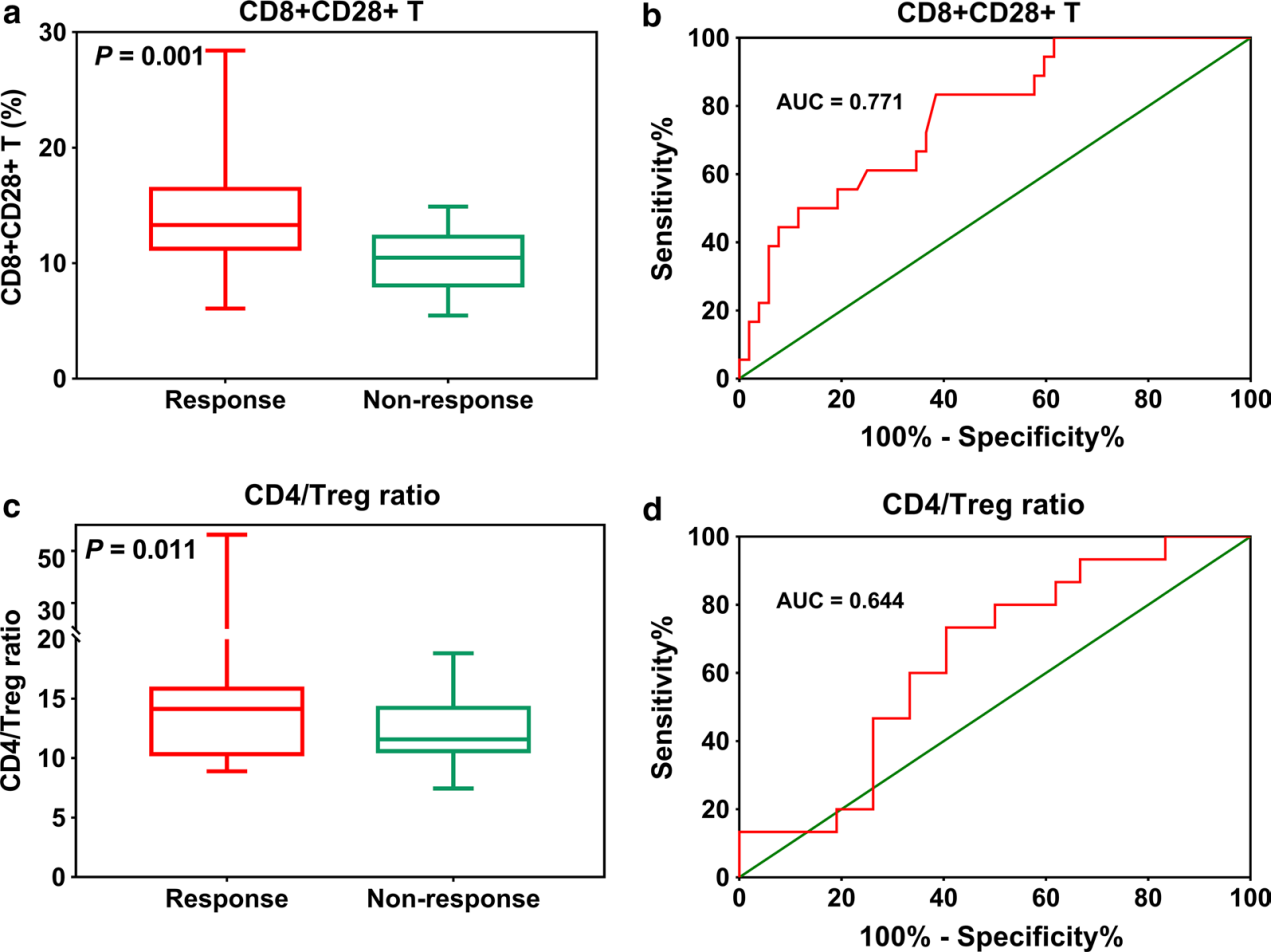
CD8+CD28+ T-cell counts in responsive and non-responsive patients (**a**) and ROC curve for CD8+CD28+ T-cell counts to distinguish responsive from non-responsive patients (**b**). CD4/Treg ratios in responsive and non-responsive patients (**c**) and ROC curve for CD4/Treg ratios to distinguish responsive from non-responsive patients (**d**) 1 month after SABR

One-month responsive patients showed higher CD8+CD28+ T-cell counts, compared non-responsive patients (14.43 ± 0.65 vs. 10.21 ± 0.66, P = 0.001, Fig. 3a). The AUC for CD8+CD28+ T cells in the distinction between responsive and non-responsive patients was 0.771 (Fig. 3b). An increased CD4/Treg ratio was observed in 1-month responsive patients, compared with non-responsive patients (16.96 ± 1.76 vs. 11.91 ± 0.74, P = 0.011, Fig. 3c). The AUC for CD4/Treg ratio to distinguish between 1-month responsive and non-responsive patients was 0.644 (Fig. 3d).

**Fig. 4:**
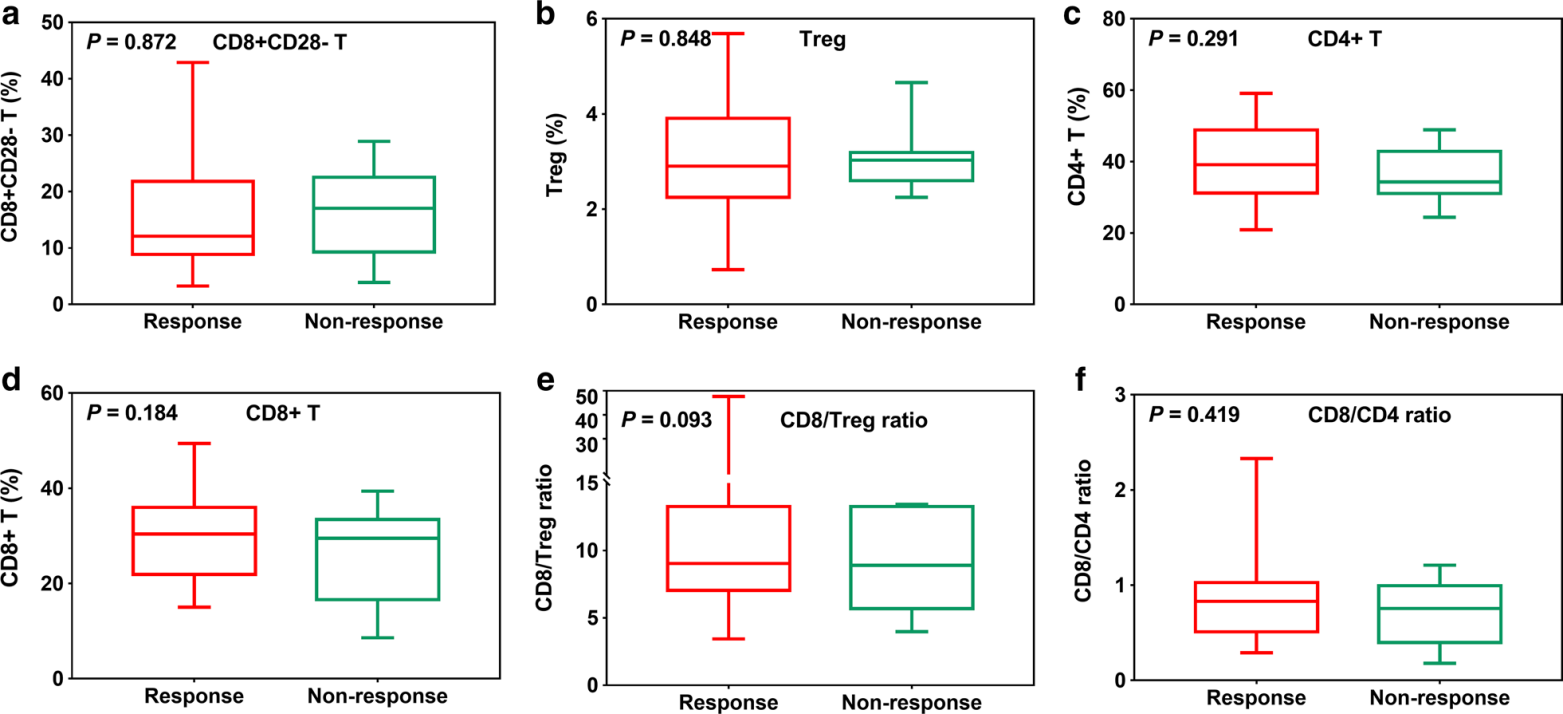
CD8+CD28− T-cell counts (**a**); Treg-cell counts (**b**); CD4+ T-cell counts (**c**); CD8+ T-cell counts (**d**); CD8/Treg ratios (**e**); and CD8/CD4 ratios (**f**) in responsive and non-responsive patients 1 month after SABR

There were no significant differences between responsive and non-responsive patients for other immune parameters 1 month after SABR (all P > 0.05, Fig. 4). ROC curves for these immune parameters to differentiate between responsive and non-responsive patients are shown in Supplementary Fig. 1. Among all the immune parameters evaluated, the most sensitive and specific marker was the CD8+CD28+ T-cell count (AUC = 0.771). The others, CD4/Treg ratio (AUC = 0.644), CD8+CD28− T-cell count (AUC = 0.532), Treg-cell count (AUC = 0.520), CD4+ T-cell count (AUC = 0.577), CD8+ T-cell count (AUC = 0.578), CD8/Treg ratio (AUC = 0.520), and CD8/CD4 ratio (AUC = 0.523), were all somewhat less sensitive and specific markers (Fig. 3, Additional file 1: Figure S1).

We reported similar results to the 1-month post-SABR treatment 6 months after SABR. Responsive patients registered higher CD8+CD28+ T-cell counts and CD4/Treg ratio, compared with non-responsive patients (P < 0.001 and P = 0.036, respectively, Additional file 1: Figure S2). The AUC for CD8+CD28+ T and CD4/Treg ratio to distinguish between responsive and non-responsive patients were 0.780 and 0.623, respectively (Additional file 1: Figure S2). There were no significant differences between responsive and non-responsive patients for other immune parameters 6 months after SABR (all P > 0.05, Additional file 1: Figure S3). The most sensitive and specific marker to differentiate responsive from non-responsive patients 6 months after SABR was also the CD8+CD28+ T-cell count (AUC = 0.780); the other immune populations were less sensitive and specific (Additional file 1: Figure S4).

### Predictive value of the CD8+CD28+ T-cell count for early tumor response to SABR

By employing the ROC curve analysis, we identified cut-off values for immune parameters (high vs. low) to distinguish tumor response from non-response. The respective cut-off values for the CD8+CD28+ T-cell count, CD4/Treg ratio, CD8+CD28− T-cell count, Treg-cell count, CD4+ T-cell count, CD8+ T-cell count, CD8/Treg ratio, and CD8/CD4 ratio were 12.52, 12.88, 12.50, 2.91, 38.80, 30.10, 8.91, and 0.79.

Table 2 shows the findings from univariate and multivariate analyses of the likelihood of an early tumor response 1 month after SABR. In the univariate analyses, high CD8+CD28+ T-cell counts (OR 0.12, 95% CI 0.03–0.48; P = 0.003), CD4/Treg ratio (OR 0.24, 95% CI 0.06–0.90; P = 0.035), and BED_10_ (OR 0.91, 95% CI 0.84–0.99; P = 0.032) predicted tumor response to SABR. We did not find statistically significant correlations between tumor response and multiple parameters: CD8+CD28− T-cell counts, Treg-cell counts, CD4+ T-cell counts, CD8+ T-cell counts, CD8/Treg ratio, and CD8/CD4 ratio (all P > 0.05). We also found no significant association between tumor response and clinicopathological characteristics, including sex, age, primary T stage, primary N stage, primary AJCC stage, histology, performance status, and smoking history (all P > 0.05).

**Table 2:** Univariate and multivariate analyses of the likelihood of early tumor response 1 month after SABR

Factors	OR	95% CI	P
CD8+CD28+ T			
Low	Reference		
High	0.12	0.03–0.48	0.003
CD8+CD28+ T (adjusted)	0.19	0.04–0.90	0.037
CD8+CD28− T			
Low	Reference		
High	1.83	0.61–5.47	0.277
Treg			
Low	Reference		
High	1.65	0.49–5.46	0.412
CD4+ T			
Low	Reference		
High	0.54	0.18–1.62	0.277
CD4/Treg ratio			
Low	Reference		
High	0.24	0.06–0.90	0.035
CD4/Treg ratio (adjusted)	0.25	0.06–1.05	0.059
CD8+ T			
Low	Reference		
High	1.00	0.34–2.92	1.000
CD8/Treg ratio			
Low	Reference		
High	0.79	0.24–2.59	0.704
CD8/CD4 ratio			
Low	Reference		
High	0.74	0.25–2.17	0.585
Age	1.03	0.98–1.09	0.140
Sex			
Female	Reference		
Male	0.97	0.31–3.03	0.960
Primary T stage			
T1	Reference		
T2–4	1.05	0.32–3.47	0.931
Primary N stage			
N0	Reference		
N1–3	1.26	0.38–4.12	0.699
Primary stage			
I	Reference		
II–III	2.66	0.53–13.18	0.229
Histology			
SCC	Reference		
AD	1.26	0.43–3.69	0.672
Performance status			
0	Reference		
1–2	0.85	0.29–2.50	0.778
Smoking history			
Non-smoker	Reference		
Smoker	0.57	0.19–1.70	0.318
Metastatic status			
Isolated lung metastasis	Reference		
Multiple metastasis	1.66	0.51–5.38	0.393
The diameter of targeted lung metastases	0.98	0.64–1.49	0.932
BED_10_	0.91	0.84–0.99	0.032
BED_10_ (adjusted)	0.90	0.80–1.02	0.109

CD8+CD28+ T-cell counts, CD4/Treg ratio, and BED_10_ were enrolled in the multivariate analyses. The results showed that only CD8+CD28+ T-cell counts independently predicted early tumor response 1 month after SABR with statistical significance (OR 0.19, 95% CI 0.04–0.90; P = 0.037). CD4/Treg ratio correlated with early tumor response with a clear trend (OR 0.25, 95% CI 0.06–1.05; P = 0.059).

To confirm the independent predictive value of immune cells, we conducted univariate and multivariate analyses of the likelihood of early tumor response 6 months after SABR and presented the results in Additional file 1: Table S1. The independent predictive value of the CD8+CD28+ T-cell count in predicting tumor response was confirmed 6 months after SABR (OR 0.08, 95% CI 0.01–0.85; P = 0.039, Additional file 1: Table S1).

## Discussion

CD28 is a co-stimulatory molecule that is required for CD8+ T cells to develop an anti-tumor response [33, 42–44]. A recent study revealed that exhausted CD8 T cells are rescued by PD-1-targeted therapies in a CD28-dependent manner [33]. In contrast, the loss of CD28 expression causes CD8 T cells to lose cytotoxic function and inhibits T cell proliferation [44]. Thus, we focused on the role of CD8+ T cells, CD8+CD28+ T cells, CD8+CD28− T cells, and other immune cells in patients with lung metastases undergoing SABR. To the best of our knowledge, the present study is the first of its kind to investigate the relationship between peripheral CD8+CD28+ T-cell count and early tumor response to SABR. We found higher peripheral CD8+CD28+ T-cell counts in patients who were responsive to SABR than in those who were non-responsive. Using logistic regression analyses, we revealed the independent predictive value of the CD8+CD28+ T-cell count for early tumor response to SABR.

A previous study reported increased CD8+CD28− T-cell counts and decreased CD8+CD28+ T-cell counts in the peripheral blood of breast cancer patients, relative to healthy controls. Moreover, there was a favorable correlation between high CD8+CD28+ T-cell counts and survival [45]. In another study conducted in melanoma patients, lower CD8+CD28+ T cells were recorded, compared with healthy volunteers; CD8+CD28+ T cells correlated positively with the 3-year survival of 38 melanoma patients but without statistical significance, which could be explained by the limited sample size [46]. Our investigation revealed that the CD8+CD28+ T-cell count correlated positively with tumor response to SABR in patients with lung metastases, which was consistent with previous findings and the anti-tumor function of these particular immune cells.

Another unique finding in our study was the presence of an increased CD4/Treg ratio in responsive patients, relative to that in their non-responsive counterparts. Also, the CD4/Treg ratio correlated with early tumor response to SABR with a clear trend. Results from a previous report revealed that high CD4/Treg ratio correlated with longer survival in a group of patients with ovarian cancer, which is consistent with our findings [47]. Another study revealed that high Treg/CD4 ratio, but not Treg/CD8 ratio, was associated with poor survival in patients with lung adenocarcinomas [48]. Our results showed comparable correlations between CD4/Treg ratio and CD8/Treg ratio and the tumor response after SABR.

Extensive research has shown that CD4+ T cells are a markedly heterogeneous group of T cells with multiple subsets (e.g., Th1, Th2, Th17, and Treg) [49]. A high CD4/Treg ratio indicates a low ratio of Treg cells among CD4+ T cells and a high ratio of T helper cells that support anti-tumor immunity. Our results suggest that in patients with high CD4/Treg ratios, the immune response was more strongly activated after SABR, thereby resulting in improved tumor regression.

A recent study revealed that post-treatment CD8+ T cells correlated with decent survival in early-stage NSCLC patients undergoing SABR [50]. Also, CD8+ tumor-infiltrating lymphocytes have been shown to correlate with tumor response after chemotherapy in breast cancer patients and chemo-radiotherapy in rectal cancer patients [19, 51, 52]. However, we did not find differences in CD8+ T-cell counts or CD4+ T-cell counts between responsive and non-responsive patients, or the predictive values of these parameters for tumor response to SABR; this may be because these are heterogeneous groups of T cells with multiple subsets. For example, CD8+ T cells include CD8+CD28− T cells and CD8+CD28+ T cells that have contrasting immune effects [42].

Treg cells contribute to the prevalence of immunosuppressive mechanisms by inhibiting the immune response toward a variety of cancer cells [53, 54]. Several studies have revealed the adverse effect of peripheral and tumor-infiltrating Treg cells on survival and tumor response in NSCLC patients after treatment [55–57]. Our results showed no significant correlation between Treg-cell counts and tumor response to SABR; we suspect that this may be related to the limited number of samples.

The results of several studies suggest that early tumor response after treatment may be associated with the survival of cancer patients [58–62]. For example, a CR after neoadjuvant chemotherapy correlated with better survival in estrogen receptor-positive/human epidermal growth factor receptor 2-negative breast cancer [59]. According to Tao et al. [58], patients with pathological CR were found to have better survival than those without pathological CR in advanced NSCLC individuals undergoing radio-chemotherapy, and the difference between the two groups reached statistical significance in relapse-free survival. With that in mind, we proposed that 1-month tumor response to SABR could as well predict survival in NSCLC. Thus far, few predictors have been investigated to determine tumor response after SABR. High BED_10_ has been associated with better tumor control through the direct cell-killing effect of radiation [17, 63]. Per this suggestion, we found that BED_10_ correlated with better tumor response to SABR. Previous studies have also shown that the shrinkage of the lung lesion by at least 20% at the last session of SABR, combined with the mean and maximum pre-SABR standard uptake values, were predictive of complete response 6 months after SABR [16, 17]. Our investigation did identify an additional factor, the pre-SABR CD8+CD28+ T-cell count, as predictive of early tumor response to SABR.

There are limitations and possible biases in our study. First, the sample size (N = 70) was somewhat limited; more extensive studies are needed in the future. Second, although we evaluated the early tumor response 1 month after SABR for all 70 patients, we were only able to assess the response in 41 patients 6 months after SABR because some patients were followed-up in their local hospitals, and we never got the results from those follow-ups. Third, previous histories of chemotherapy, radiotherapy, and surgery may have influenced the peripheral immune cell counts in our investigation. Finally, a different radiation dose was used for lung metastases because of usual tissue constraints.

## Conclusions

Our results suggest that the pre-SABR CD8+CD28+ T-cell count predicts early tumor response to SABR in patients with lung metastases from NSCLC independently. The results also highlight the importance of patient immune status in ensuring the remarkable efficiency of SABR. Identification of patients who are not responsive to SABR could facilitate the optimization of treatment strategies, such as those including the combined administration of chemotherapy or immune checkpoint inhibitors.

## Additional file

**Additional file 1: Figure S1.** ROC curves for CD8+CD28− T-cell counts (A); Treg-cell counts (B); CD4+ T-cell counts (C); CD8+ T-cell counts (D); CD8/Treg ratio (E); and CD8/CD4 ratio (F) to distinguish responsive from non-responsive patients 1 month after SABR. **Figure S2.** CD8+CD28+ T-cell counts in responsive and non-responsive patients (A) and ROC curve for CD8+CD28+ T-cell counts to distinguish responsive from non-responsive patients (B). CD4/Treg ratios in responsive and non-responsive patients (C) and ROC curve for CD4/Treg ratios to distinguish responsive from non-responsive patients (D) 6 months after SABR. **Figure S3.** CD8+CD28− T-cell counts (A); Treg-cell counts (B); CD4+ T-cell counts (C); CD8+ T-cell counts (D); CD8/Treg ratios (E); and CD8/CD4 ratios (F) in responsive and non-responsive patients 6 months after SABR. **Figure S4.** ROC curves for CD8+CD28− T-cell counts (A); Treg-cell counts (B); CD4+ T-cell counts (C); CD8+ T-cell counts (D); CD8/Treg ratios (E); and CD8/CD4 ratios (F) to distinguish responsive from non-responsive patients 6 months after SABR. **Table S1.** Univariate and multivariate analyses of the likelihood of early tumor response 6 months after SABR.

## Abbreviations

NSCLC: non-small cell lung cancer
SABR: stereotactic ablative radiotherapy
CR: complete response
PR: partial response
SD: stable disease
ROC: receiver operating characteristic
