# Supplementary material for: Increased CD8+CD28+ T cells independently predict better early response to stereotactic ablative radiotherapy in patients with lung metastases from non-small cell lung cancer
Source: J Transl Med. 2019 Apr 11;17:120. doi: 10.1186/s12967-019-1872-9 (PMC6458628; doi:10.1186/s12967-019-1872-9)
Supplement: Supplementary file 1 — Additional file 1: Figure S1. ROC curves for CD8+CD28− T-cell counts (A); Treg-cell counts (B); CD4+ T-cell counts (C); CD8+ T-cell counts (D); CD8/Treg ratio (E); and CD8/CD4 ratio (F) to distinguish responsive from non-responsive patients 1 month after SABR. Figure S2. CD8+CD28+ T-cell counts in responsive and non-responsive patients (A) and ROC curve for CD8+CD28+ T-cell counts to distinguish responsive from non-responsive patients (B). CD4/Treg ratios in responsive and non-responsive patients (C) and ROC curve for CD4/Treg ratios to distinguish responsive from non-responsive patients (D) 6 months after SABR. Figure S3. CD8+CD28− T-cell counts (A); Treg-cell counts (B); CD4+ T-cell counts (C); CD8+ T-cell counts (D); CD8/Treg ratios (E); and CD8/CD4 ratios (F) in responsive and non-responsive patients 6 months after SABR. Figure S4. ROC curves for CD8+CD28− T-cell counts (A); Treg-cell counts (B); CD4+ T-cell counts (C); CD8+ T-cell counts (D); CD8/Treg ratios (E); and CD8/CD4 ratios (F) to distinguish responsive from non-responsive patients 6 months after SABR. Table S1. Univariate and multivariate analyses of the likelihood of early tumor response 6 months after SABR. [file 12967_2019_1872_MOESM1_ESM.docx]

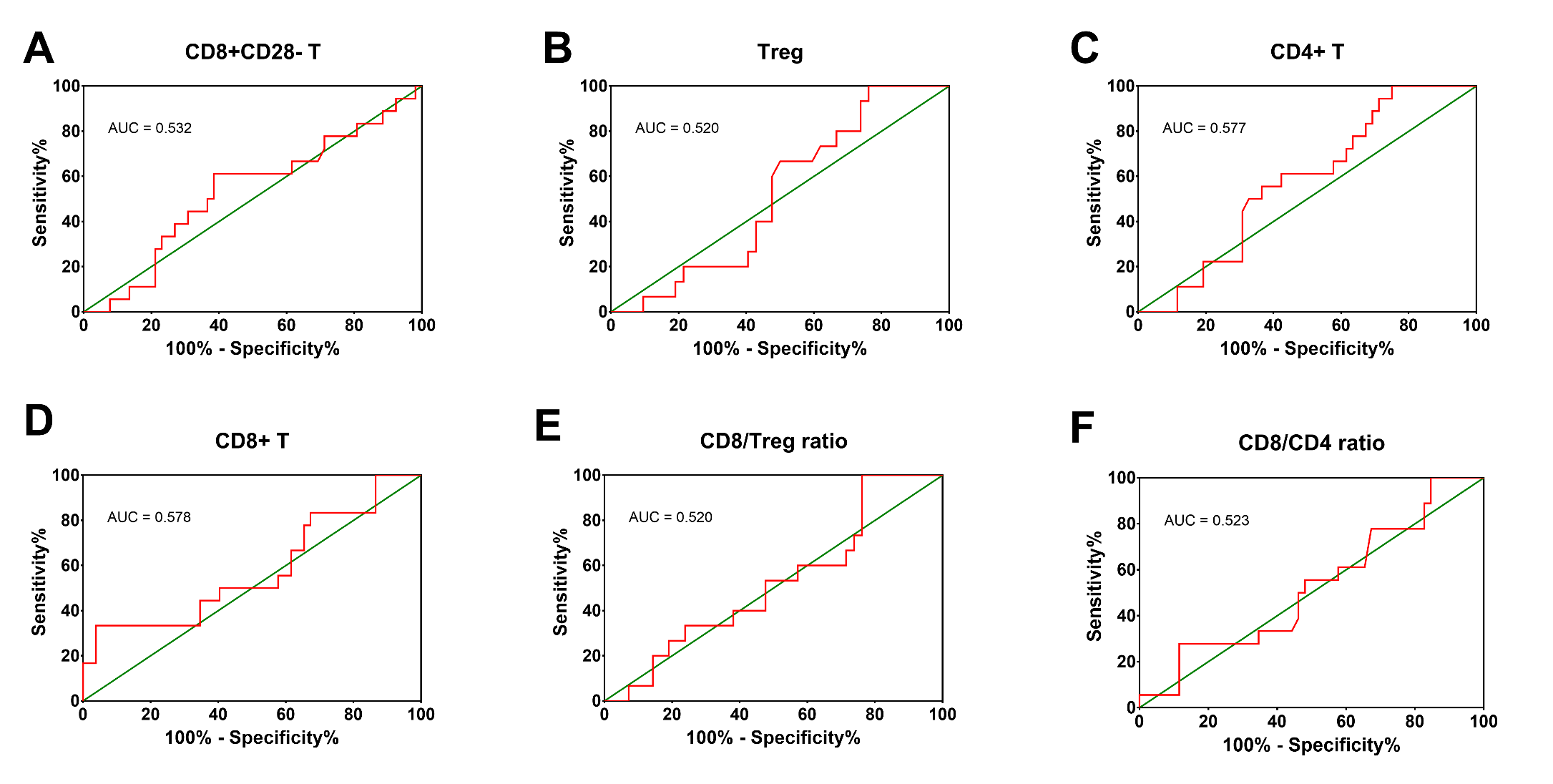


**Figure S1** ROC curves for CD8+CD28- T-cell counts (A); Treg-cell counts (B); CD4+ T-cell counts (C); CD8+ T-cell counts (D); CD8/Treg ratio (E); and CD8/CD4 ratio (F) to distinguish responsive from non-responsive patients 1 month after SABR.


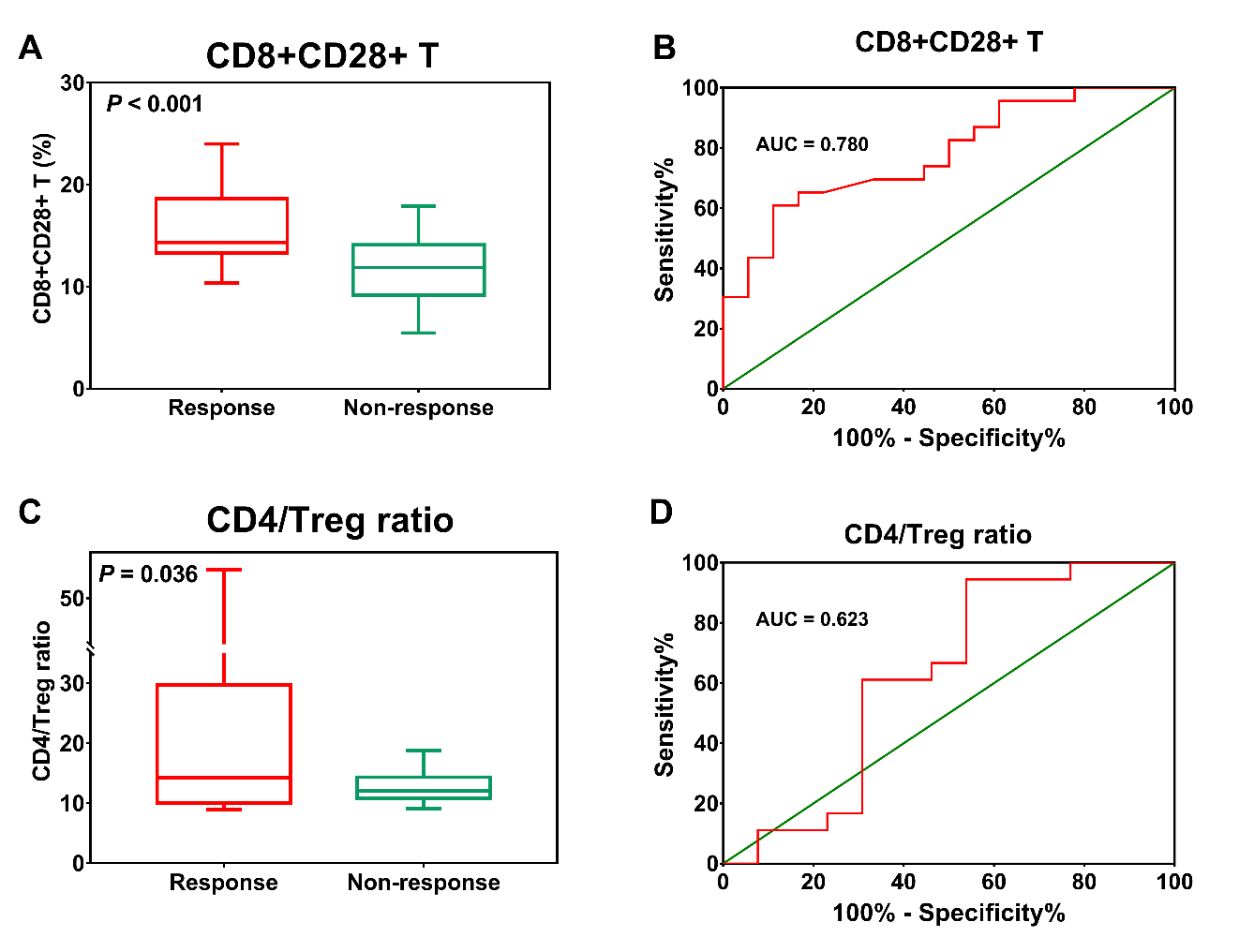


**Figure S2** CD8+CD28+ T-cell counts in responsive and non-responsive patients (A) and ROC curve for CD8+CD28+ T-cell counts to distinguish responsive from non-responsive patients (B). CD4/Treg ratios in responsive and non-responsive patients (C) and ROC curve for CD4/Treg ratios to distinguish responsive from non-responsive patients (D) 6 months after SABR.


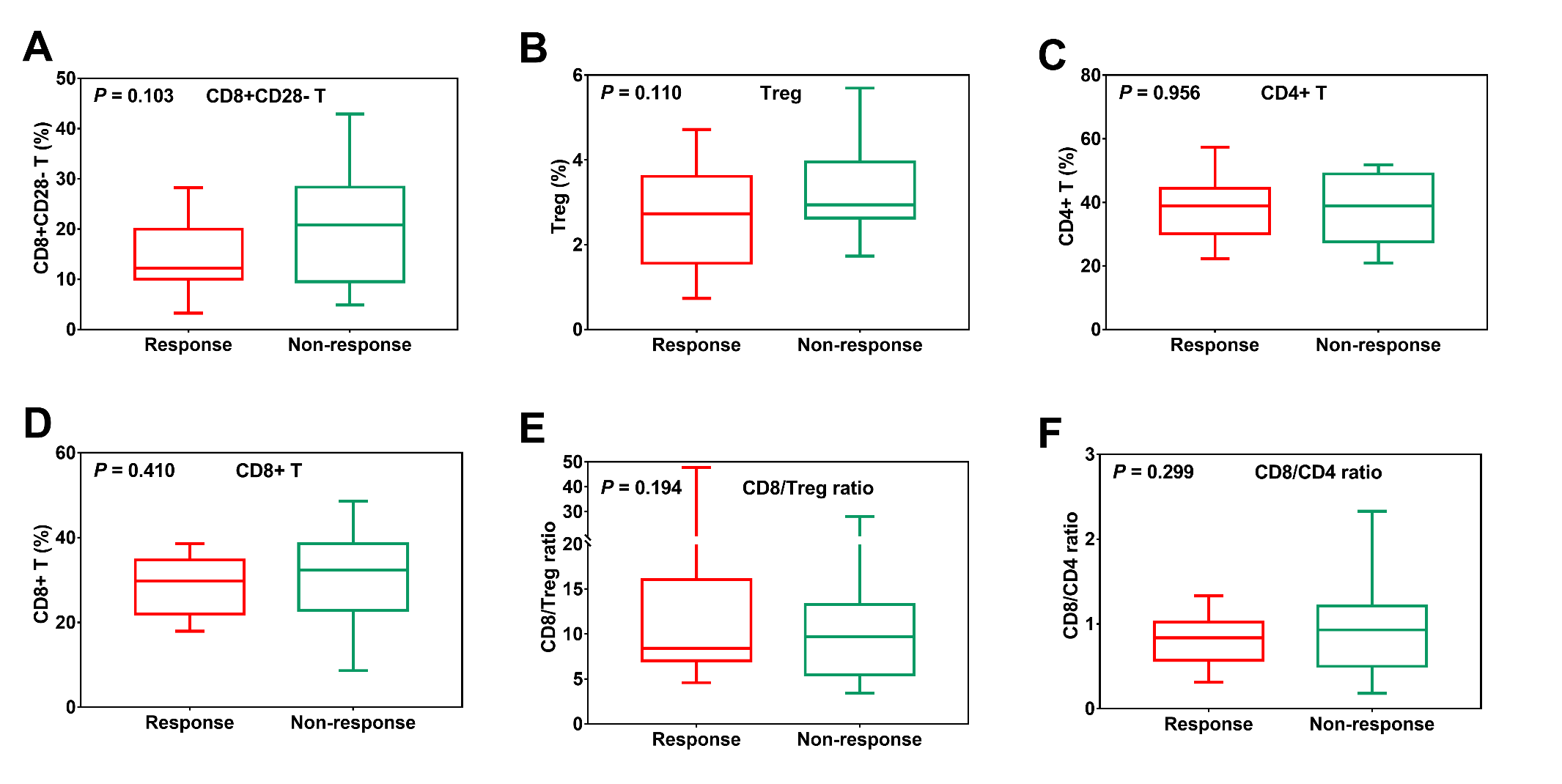


**Figure S3** CD8+CD28- T-cell counts (A); Treg-cell counts (B); CD4+ T-cell counts (C); CD8+ T-cell counts (D); CD8/Treg ratios (E); and CD8/CD4 ratios (F) in responsive and non-responsive patients 6 months after SABR.


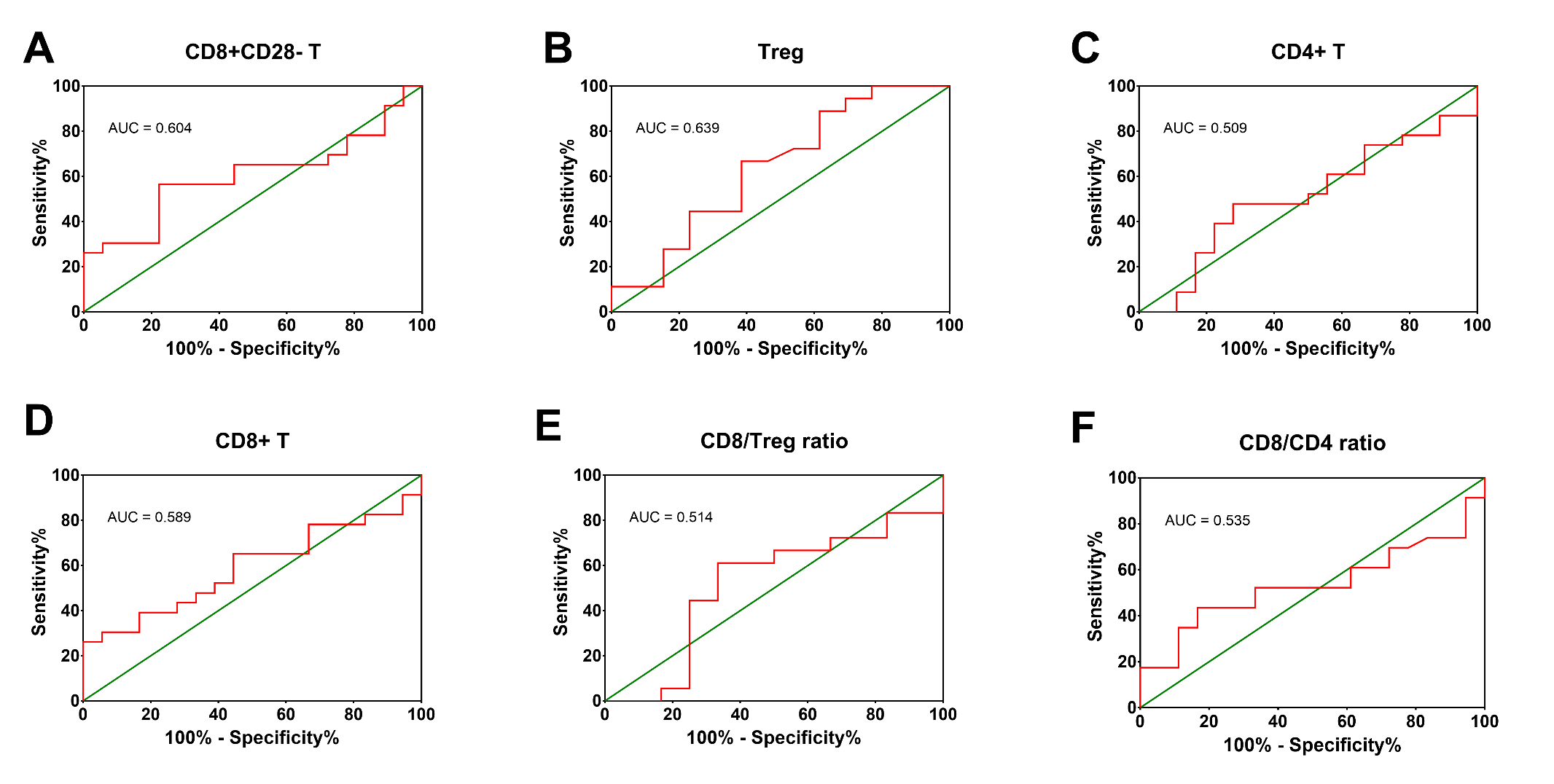


**Figure S4** ROC curves for CD8+CD28- T-cell counts (A); Treg-cell counts (B); CD4+ T-cell counts (C); CD8+ T-cell counts (D); CD8/Treg ratios (E); and CD8/CD4 ratios (F) to distinguish responsive from non-responsive patients 6 months after SABR.

**Table S1** Univariate and multivariate analyses of the likelihood of early tumor response 6 months after SABR

| Factors | OR | 95% CI | P |
| --- | --- | --- | --- |
| CD8+CD28+ T |  |  |  |
| Low | Reference |  |  |
| High | 0.09 | 0.02-0.51 | 0.006 |
| CD8+CD28+ T (adjusted) | 0.08 | 0.01-0.85 | 0.039 |
| CD8+CD28- T |  |  |  |
| Low | Reference |  |  |
| High | 2.34 | 0.66-8.30 | 0.187 |
| Treg |  |  |  |
| Low | Reference |  |  |
| High | 1.60 | 0.37-6.82 | 0.525 |
| CD4+ T |  |  |  |
| Low | Reference |  |  |
| High | 0.91 | 0.26-3.14 | 0.890 |
| CD4/Treg ratio |  |  |  |
| Low | Reference |  |  |
| High | 0.28 | 0.06-0.953 | 0.046 |
| CD4/Treg ratio (adjusted) | 0.30 | 0.05-1.87 | 0.198 |
| CD8+ T |  |  |  |
| Low | Reference |  |  |
| High | 2.34 | 0.66-8.30 | 0.187 |
| CD8/Treg ratio |  |  |  |
| Low | Reference |  |  |
| High | 2.00 | 0.46-8.55 | 0.350 |
| CD8/CD4 ratio |  |  |  |
| Low | Reference |  |  |
| High | 0.87 | 0.25-3.01 | 0.829 |
| Age | 1.02 | 0.96-1.09 | 0.411 |
| Sex |  |  |  |
| Female | Reference |  |  |
| Male | 0.87 | 0.22-3.42 | 0.853 |
| Primary T stage |  |  |  |
| T1 | Reference |  |  |
| T2-4 | 1.87 | 0.53-6.16 | 0.329 |
| Primary N stage |  |  |  |
| N0 | Reference |  |  |
| N1-3 | 3.54 | 0.95-13.19 | 0.060 |
| Primary stage |  |  |  |
| I | Reference |  |  |
| II-III | 4.75 | 1.15-19.65 | 0.031 |
| Primary stage (adjusted) | 4.62 | 0.39-25.63 | 0.221 |
| Histology |  |  |  |
| SCC | Reference |  |  |
| AD | 3.11 | 0.85-11.29 | 0.084 |
| Performance status |  |  |  |
| 0 | Reference |  |  |
| 1-2 | 0.27 | 0.08-1.26 | 0.068 |
| Smoking history |  |  |  |
| Non-smoker | Reference |  |  |
| Smoker | 0.37 | 0.09-1.48 | 0.161 |
| Metastatic status |  |  |  |
| Isolated lung metastasis | Reference |  |  |
| Multiple metastasis | 0.75 | 0.13-4.25 | 0.745 |
| The diameter of targeted lung metastases | 1.11 | 0.76-1.61 | 0.584 |
| BED_10_ | 0.92 | 0.87-0.95 | 0.040 |
| BED_10_ (adjusted) | 0.94 | 0.84-1.05 | 0.267 |
